# Integrated bioinformatics analysis and experimental validation to understand tryptophan metabolism-related genes in hepatocellular carcinoma

**DOI:** 10.7150/jca.91306

**Published:** 2024-07-16

**Authors:** Hao Chen, Wu Sun, Mengxiao Xie, Zhenhan Li, Bofeng Chen, Tao Zhang, Le Lv, Lizhen Wang, Linming Lu, Qianli Tang, Liangping Luo

**Affiliations:** 1 Guangxi Clinical Medical Research Center for Hepatobiliary Diseases, Affiliated Hospital of Youjiang Medical University for Nationalities, Baise, China.; 2 Postdoctoral Research Station of Clinical Medicine, Jinan University, Guangzhou, China.; 3 Department of Pathology, School of Basic Medical Sciences, Wannan Medical College, Wuhu, China.; 4 Department of Pathology, the First Afiliated Hospital, Yijishan Hospital of Wannan Medical College, Wuhu, China.; 5 Department of Laboratory, the First Affiliated Hospital of Nanjing Medical University, Nanjing, China.; 6 School of Clinical Medicine, Wannan Medical College, Wuhu, China.; 7 School of Public Health, Wannan Medical College, Wuhu, China.; 8 School of Nursing, Wannan Medical College, Wuhu, China.; 9 Medical Imaging Center, the First Affiliated Hospital of Jinan University, Guangzhou, China.

**Keywords:** tryptophan metabolism, ALDH2, hepatocellular carcinoma, Immune infiltration

## Abstract

**Background:** Tryptophan (Trp) metabolism is closely related to tumor immunity, and its disorder can cause an immunosuppressive microenvironment, promoting the occurrence and development of hepatocellular carcinoma (HCC). The aim of this study is to explore and validate the independent prognostic genes in patients suffered from HCC.

**Methods:** The transcriptome data of GSE87630 from GEO database were downloaded to analyze differentially expressed genes (DECs) which were intersected with the gene sets of Trp metabolism from MsigDB database. Univariate/multivariate COX regression was performed to identify the genes with independent prognostic significance. TCGA, GTEx, UALCAN, and GEPIA2 databases were applied to analyze DEGs for prognosis. RNA seq data of HCC from TCGA database were collected for Lasso regression analysis. The ssGSEA algorithm was used to perform the analysis of TCGA data. The effects of the candidate differential gene on HCC cells proliferation and migration were evaluated using EdU immunofluorescence and transwell assays.

**Results:** Trp metabolism-related DECs for HCCs were obtained, including MAOB, CYP1A2, KYNU, CYP2E1, ALDH2, CYP2C18, TDO2, AOX1, CYP3A4 and INMT. Moreover, multivariate COX regression results showed that ALDH2 can serve as an independent prognostic molecule and its transcriptional and translational levels were significantly reduced in the tumor tissues. The low expression of ALDH2 was associated with poor prognosis. Overexpression of ALDH2 dramatically reduced the HCC cells proliferation and migration.

**Conclusion:** ALDH2 is associated with Trp metabolism and its downregulation in HCC has a potential value on prognosis. Overexpression of ALDH2 can reduce the proliferation and migration of HCC cells.

## Introduction

Hepatocellular carcinoma (HCC) is the third most frequent cause of cancer-related deaths in the world, causing approximately 7 million deaths worldwide 1. Although the intervention measures involving surgical treatment, liver transplantation, radiofrequency ablation, radiotherapy/chemotherapy and immunotherapies may be effective for patients with HCC, the 5-year survival rate is only 50%-70% 2. Metabolic disorder has been implicated in several types of cancers including HCC. Thus, identifying metabolism-related biomarkers might provide new targets for HCC prognostic evaluation as well as targeted therapies.

Tryptophan (Trp) is closely related to various nutrients such as carbohydrates, proteins, fats, vitamins and trace elements in the metabolic process, mainly from the diet. Trp metabolism is involved in manipulating immunity, neuronal function, and intestinal homeostasis through kynurenine pathway (KP). The imbalance of Trp metabolism in diseases ranging from cancer to neurodegenerative diseases has sparked interest in the therapies targeting KP 3. In pancreatic cancer, it has been proved that Trp can provide a nutritional source for folate dependent single carbon cycling, causing tumor cell proliferation 4. Meanwhile, the changes in Trp metabolism can occur in the early stages of colorectal cancer, serving as an adaptive mechanism for tumors to evade immune surveillance and to occur metastasis, and diet-regulated Trp metabolism may serve as a treatment option for colorectal cancer 5. Kynurenine-3-monooxygenase (KMO) is a very important enzyme in KP, which can catalyze the hydroxylation of KYN to 3-HK, producing free radicals and leading to cell apoptosis. *In vitro* experiments showed that compared with the normal liver cells, KMO enzyme levels are upregulated in HCC cells. KMO may play a role in promoting tumor proliferation, metastasis, and invasion, and knockdown of KMO enzyme can reduce cancer cell proliferation. KMO may be a new target for HCC treatment 6.

Currently, the researches on Trp metabolism in diseases are limited to general bulk sequencing and serum metabolites 7-10. In this research, how the genes related Trp metabolism affect HCC occurrence and development was investigated furtherly at the single-cell level in this study accompanied by the experimental verification.

## Materials and Methods

### Bioinformatics analysis

#### GEO data acquisition

The transcriptome data of GSE87630 from GEO database (http://www.ncbi.nlm.nih.gov/projects/geo) were downloaded for analysis, including 64 tumor samples and 30 normal samples as previously reported 11. R and R packet limma were used to perform DEG analysis, log2FC >1.5 and* P*.adj<0.05 as the threshold for screening differential genes. PCA and box normalization charts were used to display the grouping information and differences between sample groups. Volcano maps were applied to display differential genes that meet the threshold. A heat map can display the genes with the greatest differences.

#### Trp metabolism genes

Trp metabolism genes were downloaded from the MsigDB database which were intersected with the previously obtained differential genes to obtain the key DEGs of Trp metabolism.

#### Lasso Regression exploring the Risk sore of key genes in Trp Metabolism

The level 3 HTSeq-FPKM format RNAseq data in the LIHC (hepatocellular carcinoma) project from TCGA (https://portal.gdc.cancer.gov/) were downloaded, and the FPKM (Fragments Per Kilobase per Million) format RNAseq data were converted into TPM (transcripts per million reads) format with log2 conversion. The transformed TCGA data were then used for various subsequent analyses with the glmnet package [version 4.1-2] and the survival package [version 3.2-10]. The prognosis type was reflected by overall survival and 10-fold cross-validation was used. The Lasso variable trajectory variable graph was used to visualize the variation of variable coefficients with the lambda value of the penalty term coefficient of Lasso. The risk factor map was sorted according to the size of the risk score.

#### Univariate and Multivariate analysis of COX regression

To explore whether the key genes in Trp metabolism have an impact on the patient prognosis and whether there are independent prognostic factors. The single factor/multi factor COX regression analysis were conducted on the TCGA data after previous conversion. The prognostic data were obtained from the reported paper 12.

#### Prognostic analysis for the independent prognostic factors

Through COX regression testing, the molecules were identified that can serve as independent prognostic factors. TCGA data converted by log2 were processed using the R package survivor package [version 0.4.9] for visualization and the survival package [versions 3.2-10] for the statistical analysis of survival data. The groups were divided according to the different clinical variables and the subgroup survival curves were drawn. The basic situation of each study object was described by the baseline data table. A nomogram was drawn based on multivariate regression analysis. In addition, a prognostic calibration chart was constructed as fitting analysis between the model established by Cox regression method and the actual situation, while also demonstrating the accuracy of the nomogram model 13.

#### Differential expression analysis of independent prognostic factors

UCSC XENA (https://xenabrowser.net/datapages/) RNAseq data in TPM format of TCGA and GTEx were processed uniformly 12. LIHC (hepatocellular carcinoma) data in TCGA and the corresponding liver normal tissue data in GTEx were extracted. The expressions were compared between the samples after converting the RNAseq data in TPM format into log2. At the same time, the multiple different databases were used to validate the differences in independent prognostic factors. The transcriptional level differences were obtained under different grouping conditions from UALCAN database with the proteomic analysis between hepatocellular carcinoma and the normal tissues. The immunohistochemical images of HCC and normal tissues were obtained in HPA database. The differences of transcriptional levels in different pathological stages were obtained from GEPIA2.

#### Exploring the relationship between independent prognostic factors and tumor microenvironment

The ssGSEA algorithm of the GSVA package [1.34.0 version] was used to perform immune infiltration level analysis for TCGA data. The markers of 24 immune cells were obtained from the data of the previously reported paper 14. Additionally, the estimate package was utilized to calculate StromalScore, ImmunoScore, and ESTIMATEScore 15.

#### Single cell sequencing reveals the relationship between independent prognostic factors and immune cell heterogeneity

The expression levels of independent prognostic factors in different cell types were explored using TISCH, and conducted GSEA, cell communication, and transcription factor enrichment analysis. The R software package Cellchat was used for the analysis of intercellular communications.

### Experimental validation

#### Cell culture

HepG2 and HCC-LM3 HCC cell lines were obtained from iCell Bioscience Inc (Shanghai, China). The cell lines were cultured in DMEM supplemented with 10% FBS, 100 U/ml penicillin, and 100 µg/ml streptomycin.

#### Transfection and viral infection

HEK293T cells were overexpressed with ALDH2 using the lentivirus ALDH2 (or GFP) plasmid. Lipofectamine2000 (plasmid: Lipo2000 1:4) was utilized as a transfection reagent. The culture medium was changed after 4-6 hours. Then, we collected lentivirus culture medium 48 hours later to infect HCC cells. Fenvalerate (10 μg/ml) was used to screening stable transfected cells. Then, the cells were amplified and collected for the subsequent analysis.

#### Western blotting

Whole cells were lysed by RIPA lysis buffer. The proteins were dissociated by SDS-PAGE and transferred to PVDF membranes. The membranes were conjugated with the antibodies as described before 16. The signals were detected with the ECL system (Pierce) and quantified by scanning densitometry with the Image Lab analysis system. The primary antibodies used in this study were as follows: ALDH2 (1:1000, Abcam, 133306), GAPDH (1:1000, Abcam, ab8245).

#### Transwell assay

Using serum-free culture medium, the cells transfected for 24 hours were prepared into single cell suspensions, with 200 μL added to each well (including 4 × 10^4^ cells); Then, we added culture medium containing 10% FBS in the lower chamber 800 μ L. After 24 hours of cultivation, the upper chamber culture medium was discarded, we then wiped off the upper layer cells of the filter membrane with a cotton swab, washed with PBS for three times, fixed the upper chamber in 4% paraformaldehyde and stained cells with 3% crystal violet. Finally, we observed the results with an inverted microscope, randomly selected 5 fields of view, counted the number of cells passing through the membrane.

#### EdU immunofluorescence

EdU staining was performed based on the protocols (Sigma-Aldrich) as previously described 17. The HCC cells were incubated by EdU solution and fixed by 4% paraformaldehyde, then 2% glycine was added, the cells were washed by PBS and added with penetrant for permeabilization, and the following steps were conducted with the guide of the EdU kit direction.

#### Statistical analysis

SPSS 22.0 software was used, and GraphPad Prism software was used for image drawing. The measurement data were expressed as mean ± standard deviation, and difference between the two groups was subjected to unpaired students' tests. The results of experimental validation were based on three independent experiments. *P*<0.05 indicated a statistically significant difference.

## Results

### Testing sample quality and obtaining the differentially expressed genes

**Figure 1:**
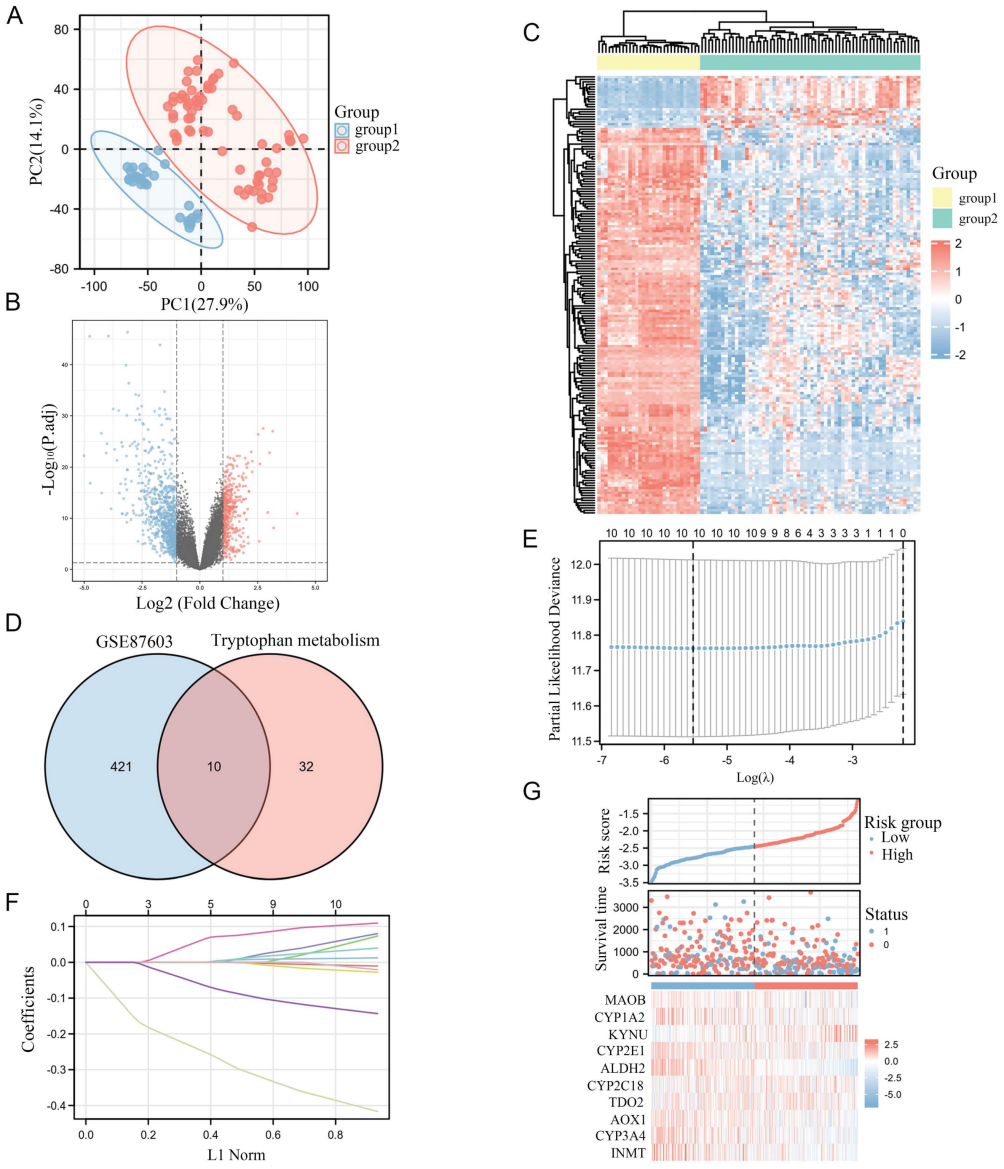
** Identification of prognostic genes related to Trp Metabolism in HCC.** A: PCA chart test for sample differences between Groups. B: The volcano map displays differential genes. C: The heat map displays the expression profile of differential genes. D: The Venn diagram shows the intersection of differentially expressed genes and Trp metabolism genes. E: Trajectory plots of different variables in Lasso. F: Lasso coefficient screening. G: Correlation between screened variables and risk score.

As the PCA diagram, 94 samples were grouped according to tumor and normal conditions, and there was a significant difference between the groups without intersection (Figure 1A). As the normalized box plot, the chip signal strength of 94 samples was basically at the same level, and there were no batch effects. The volcano map indicated that 431 DEGs met the threshold, while the heat map was used to visualize the matrix data corresponding to the DEGs (Figure 1B-C).

### Intersection of Trp metabolism genes and the differentially expressed genes

42 genes in Trp metabolism were added and intersected with the DEGs that met the conditions, and 10 differentially expressed Trp metabolism-related genes were obtained (Figure 1D). Afterwards, we constructed a Lasso regression model based on the clinical data from TCGA and calculated the Risk score (Figure 1E). The Lasso coefficient screening indicated that all 10 genes were with the certain prognostic significance, and the trajectory variable graph was used to visualize the changes in the coefficients of the ten variables with the lambda value of the penalty term coefficient of Lasso (Figure 1F). In order to identify the prognostic gene for Trp metabolism, we conducted univariate/multivariate COX regression and found that ALDH2 can serve as an independent prognostic molecule (Table 1). The metabolim-related genes had certain correlations with the Risk score according to the risk factor graph. With the increasing of Risk score, the expression levels of ALDH2 decreased with poor prognosis (Figure 1G).

### Identification of differentially expressed ALDH2 and its clinical value

**Figure 2:**
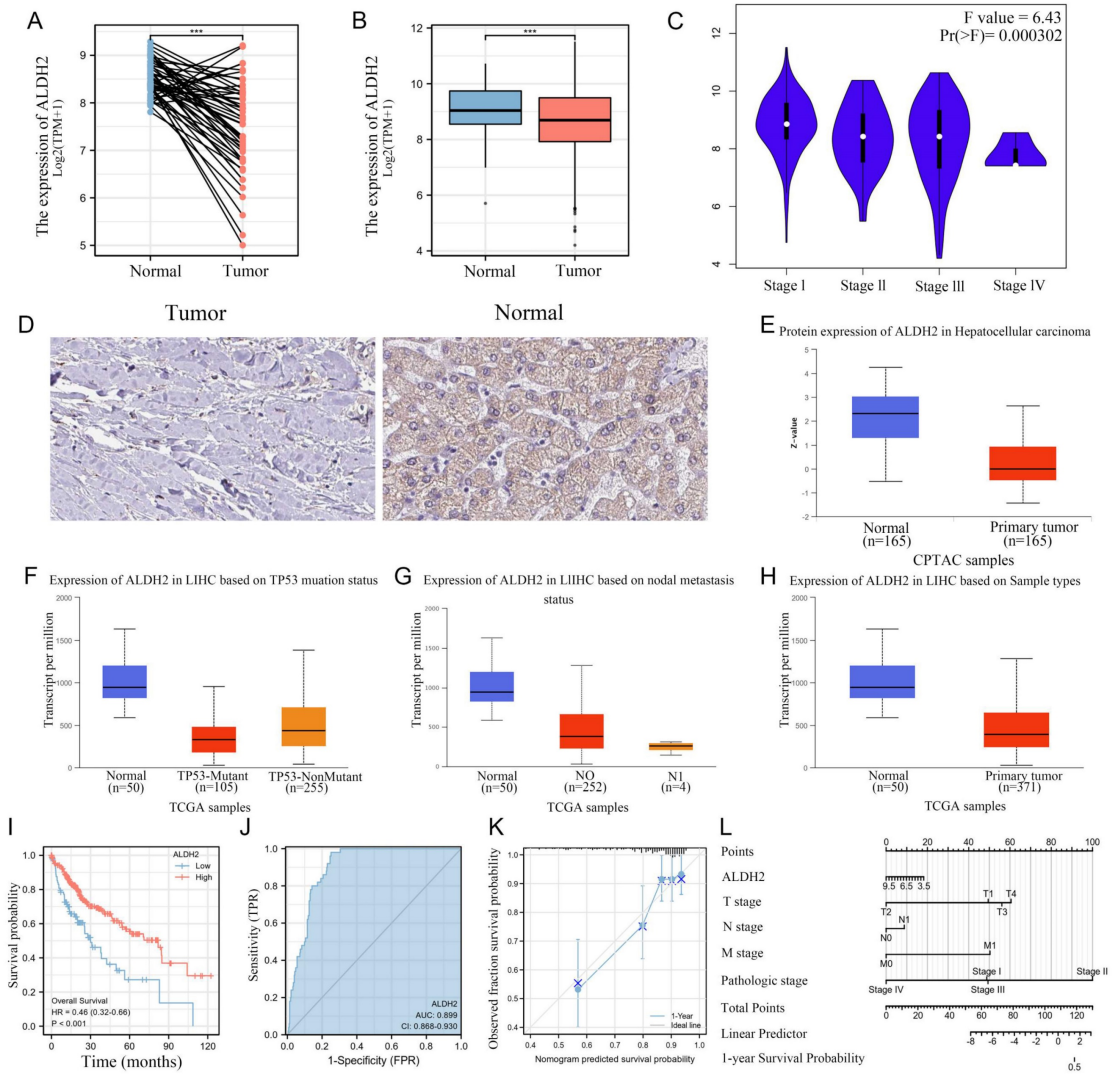
** ALDH2 can be used as an independent prognostic molecule for HCC.** A: Differential expression of ALDH2 in paired samples. B: Differential expression of ALDH2 in unpaired samples. C: The expression differences of ALDH2 in different pathological stages. D: Immunohistochemistry demonstrated the protein difference of ALDH2 between tumor and normal tissues. E: UNLCAN demonstrated a significant decrease in ALDH2 protein levels in tumors. F: The relationship between ALDH2 expression and TP53 mutation status. G: The relationship between ALDH2 expression and lymph node metastasis. H: Differential expression of ALDH2 in UALCAN. I: The relationship between the expression level of ALDH2 and prognostic outcomes. J: The ROC curve demonstrated the diagnostic value of ALDH2. K-L: Nomogram and Calibration plots indicated that ALDH2 was with good stability as a prognostic molecule.^ ***^P<0.001.

We found that the transcription level of ALDH2 was significantly reduced in tumors from both non paired and paired samples. Meanwhile, ALDH2 mRNA level intensively decreased with the progression of the pathological stage (Figure 2A-C). Meanwhile, immunohistochemistry also showed a significant reduction in ALDH2 protein abundance in tumors, which was confirmed by data from CPTAC (Figure 2D-E). We found that the mRNA level of ALDH2 is much lower than that in the TP53 wildtype, and the lower the expression of ALDH2 was, the more lymph node metastasis occured in HCC. Then, the low expression of ALDH2 in tumors was validated in UALCAN (Figure 2F-H). In order to explore the prognostic value of ALDH2 in HCC, we plotted a survival curve based on the expression level of ALDH2 with a prognosis type of OS. The results showed that patients in the group with lower ALDH2 expression had poorer prognosis outcomes (HR=0.46, *P*<0.001). In order to identify the diagnostic value of ALDH2, we plotted an ROC curve and observed an AUC of 0.899, indicating a high diagnostic value. More importantly, we established a model using the multi factor Cox regression method and presented it using nomogram diagrams. The prognosis calibration chart is mainly used for fitting analysis between the model established by Cox regression method and the actual situation. It can be seen that the predicted line and diagonal line are very close, indicating that the model fitting effect is better (Figure 2I-L).

### ALDH2 and immune infiltration

**Figure 3:**
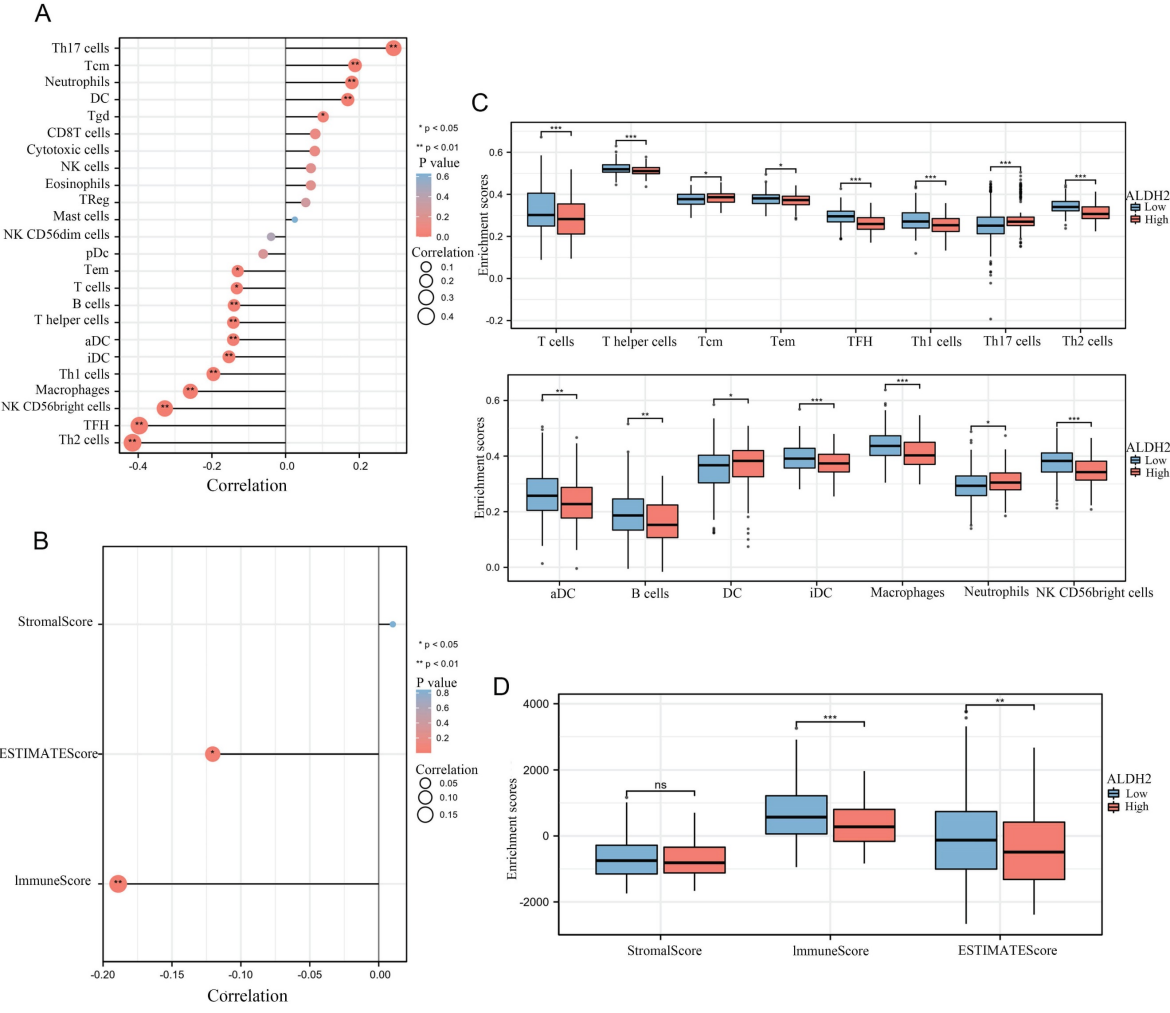
** ALDH2 was associated with the immune infiltration in HCC.** A: The ssGSEA algorithm calculated the correlation between the expression levels of ALDH2 and different levels of immune cell infiltration. B: The ESTIMATE algorithm calculated the relationship between ALDH2 and immune score. C-D: Comparing the differences in immune cell infiltration levels among different groups based on ALDH2 expression levels. ^*^P<0.05, ^**^P<0.01, ^***^P<0.001.

We calculated the correlation between the expression of ALDH2 and the infiltration levels of 24 different immune cells and found that a total of 16 immune cells were significantly correlated with ALDH2. It can be observed that the expression level of ALDH2 was significantly positively correlated with the infiltration score of Th17 cells but negatively correlated with the infiltration score of Th2 cells (Figure 3A). In addition, we also used 'Estimate' to calculate the matrix score and immune score and found a significant negative correlation between ALDH2 expression and ImmunoScore as well as ESTIMATEScore (Figure 3B). In the group comparison chart, it can be seen that most immune cell infiltration significantly increased in the group of ALDH2 with lower expression. Similarly, ImmunoScore and ESTIMATEScore had higher scores in the ALDH2 low expression group, while StromalScore showed no significant difference. The scatter plot also displayed a strong negative correlation between the infiltration level of many immune cells and the expression levels of ALDH2 (Figure 3C-D).

### Single cell sequencing revealed the specific expression of ALDH2

**Figure 4:**
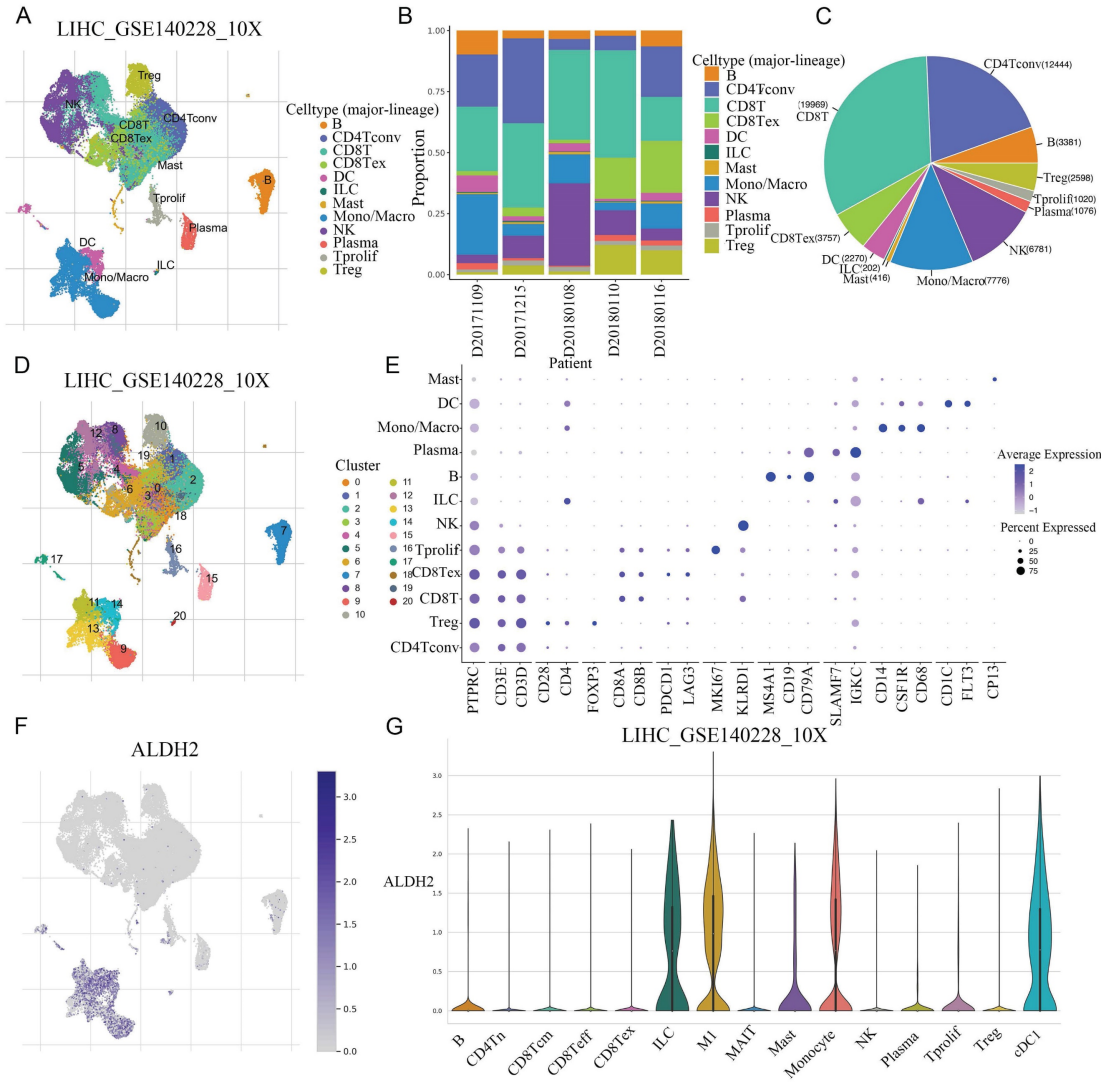
** The specific expression of ALDH2 were explored in different cell types at single-cell level.** A: After dimensionality reduction clustering of single cell data in GSE140228, a total of 21 different clusters were obtained. B: The proportion of different cell types in the 5 samples. C: The pie chart showed the number of cells included in different cell types. D: Subdivide the cell population into 15 different cell subpopulations. E: The cell markers and expression levels used to annotate cell types. F: The UMAP graph showed the expression level of ALDH2. G: The violin diagram showed the expression levels of ALDH2 in different cell types.

The common NGS usually only detects the average expression level of genes within tissues, while ignoring the heterogeneity of different tissues. Single cell sequencing can reduce dimensionality and cluster a large number of cells to obtain cell subpopulations. After conducting dimensionality reduction clustering analysis on single cell data in GSE140228, a total of 21 different clusters were obtained. Through cell marker annotation, 12 different cell types were classified, with CD8^+^T cells accounting for the largest proportion (Figure 4A-C). Further the 12 cell types subdivided into 15 different cell types (Figure 4D). The cell markers and expression used for each cell typing were also shown (Figure 4E). The UMAP map showed a significant and specific increase in ALDH2 expression in monocytes/macrophages. In the violin plot, the expression levels of ALDH2 in various cell subtypes can be observed, with a significant upregulation in M1 macrophages and a higher expression level in DCs compared to other cell types (Figure 4F-G).

### Cell communication, and transcription factor analysis

**Figure 5:**
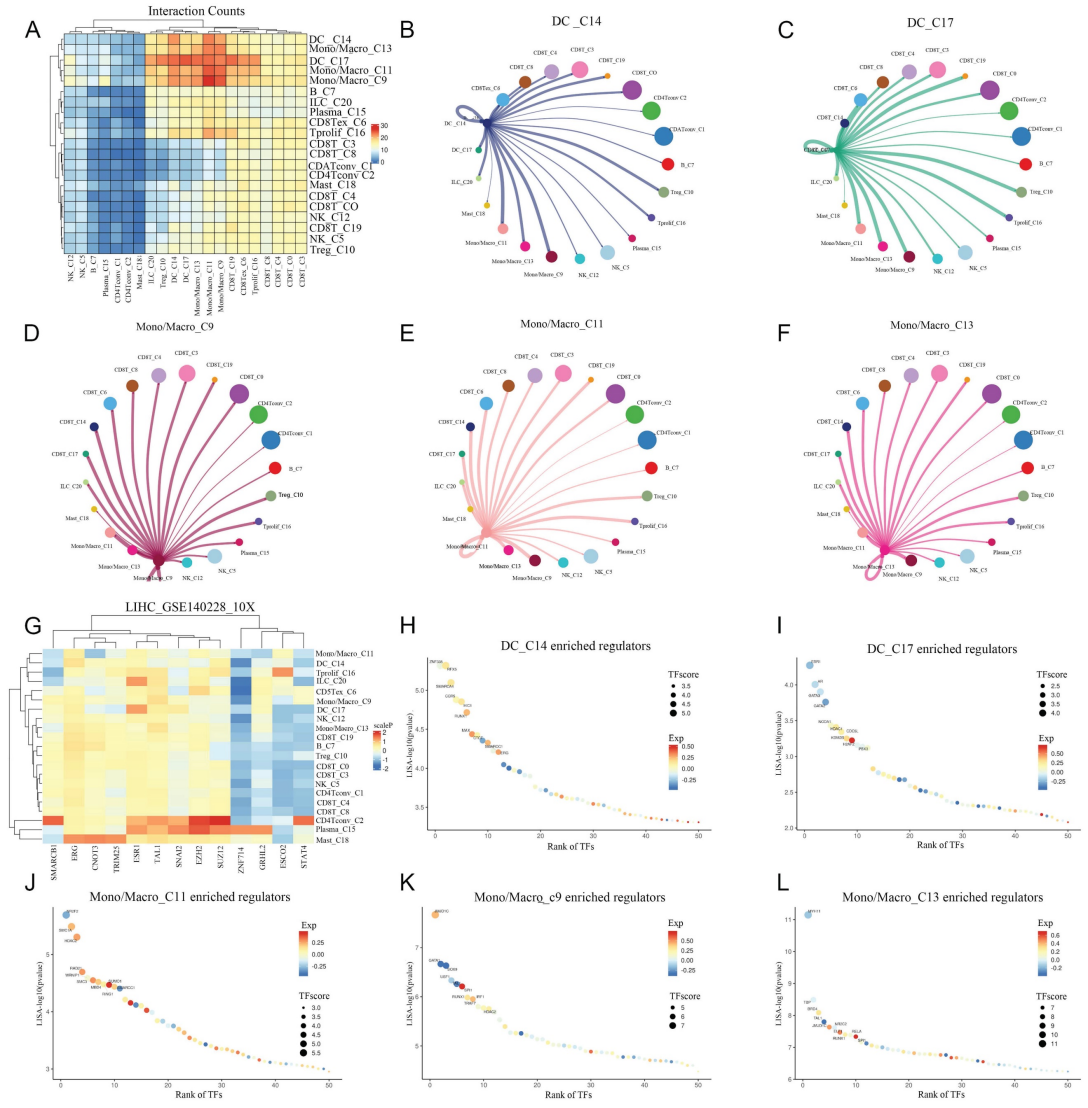
** Analysis of intercellular communication and cis regulation.** A: The heat map showed the intensity of cell interaction between different types of cells. B-C: circle diagram showed the strength of communication between DC and other cells. D-F: circle diagram showed the intensity of communication between Mono/Macro cells and other cells. G: Transcription factors regulating intensity heat maps in different cell types. H-I: Ranking diagram of the most significant transcription factors in DC cells. J-L: Ranking diagram of the most significant transcription factors in Mono/Macro cells.

**Figure 6:**
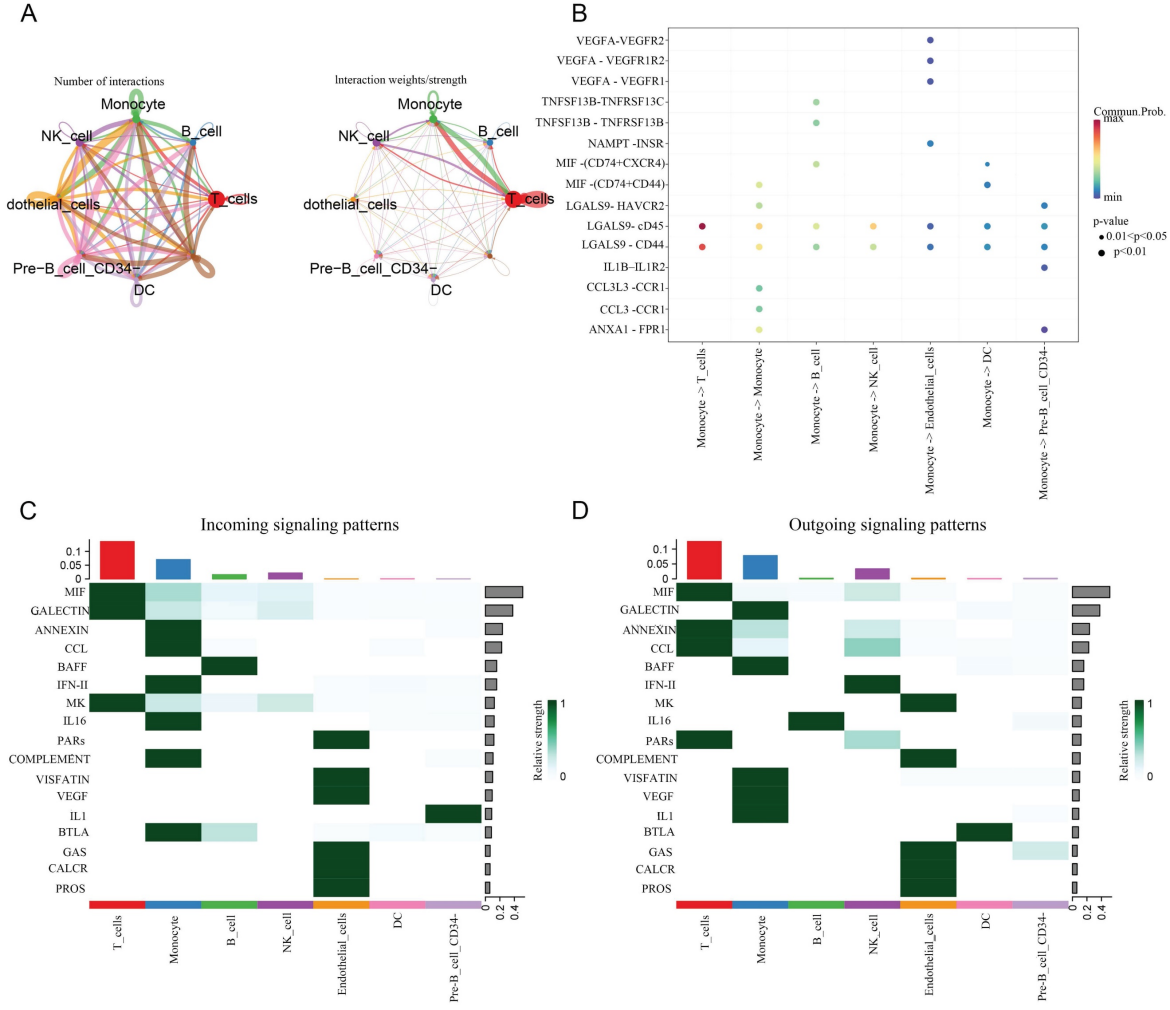
** The levels of cellular communication between the seven major cell populations.** A: The number and intensity of interactions between cell populations. B: Starting from monocytes, the receptor ligand pairs connected by intercellular communication with various cell populations, and their strength. C: Income. D: Outgoing.

The communication information between different cells in HCC has not yet been fully elucidated. So, we delved deeper into the communication between cell types we previously obtained, and found that in addition to strengthening communication with themselves, monocytes/macrophages had much stronger communication with DCs compared with other types of cells. The direct communication levels between NK cells, B cells, Plasma, CD4^+^T cells, mast cells and other cells were significantly reduced (Figure 5A). We explored the communication between DCs and monocytes/macrophages with autologous and other cells. It was found that in DCs, in addition to having strong communication signals with themselves, the intensity of cell interaction with monocytes/macrophages is also high. In addition, DC also has strong cell interaction with Treg and Tprolif (Figure 6B-C). In monocytes/macrophages, in addition to maintaining strong interactions with themselves, they also keep strong interactions with CD8T cells, DC cells, Treg, and Tprolif cells (Figure 5D-F). In order to decipher the reconstruction of gene regulatory networks and cell states at the single-cell level, we conducted cis regulatory analysis to guide the recognition of transcription factors and cell states. By constructing a cis regulatory network for transcription factor regulation levels and cell types, we found that STAT4 and ZNF714 can significantly regulate DCs, and monocytes/macrophages also exhibit a similar regulatory relationship to DCs (Figure 5G). In DC_C14, the most significant one is ZNF335, the most significant one is ESR1 in in DC_C17 (Figure 5H-I). Among monocytes/macrophages, the most significant ones are JMJD1C, NR2F2 and MYH11 (Figure 5-L).

Afterwards, we further explored the signaling pathways between cell communication in several major cell populations with Cellchat. We found that the interaction weight/strength between monocytes and T cells was significantly stronger than other cell populations. In addition, it was also shown that the communication between LGALS9-CD44/CD45 ligand receptor ligation occurred from monocytes to all other cell populations, especially between T cells. More importantly, we demonstrated the coordinated patterns of 17 signaling pathways between multiple cell types through heat maps (Figure 6).

**Figure 7:**
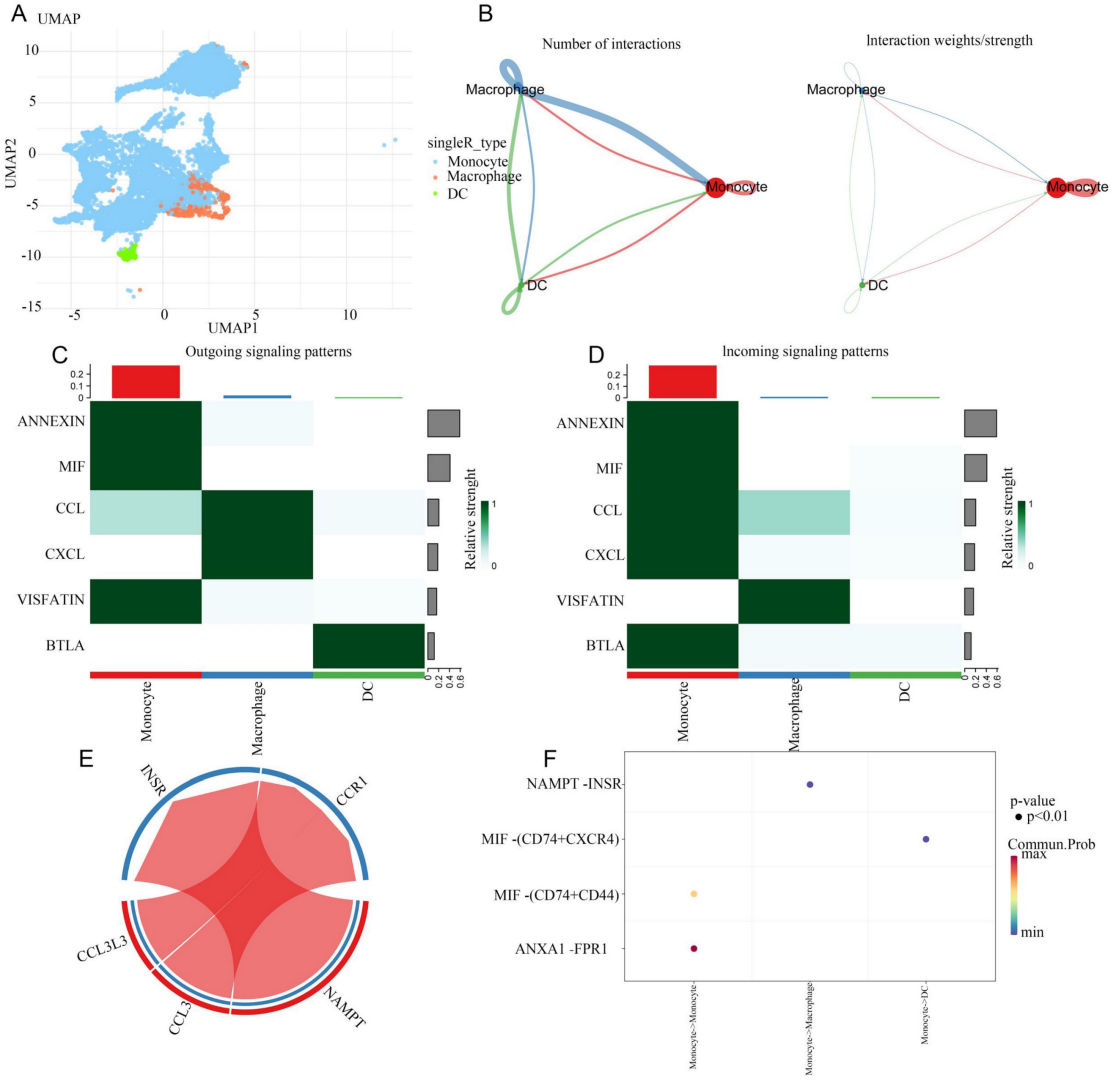
** Extracted cell subpopulations for further analysis.** A: UMAP map of beautified cell subpopulations. B: The number and intensity of interactions between cell populations. C: Outgoing signaling patterns from monocytes. D: Incoming signaling patterns to monocytes. E: Network visualization of signal molecules in monocytes. F: Significance and protein interaction analysis.

**Figure 8:**
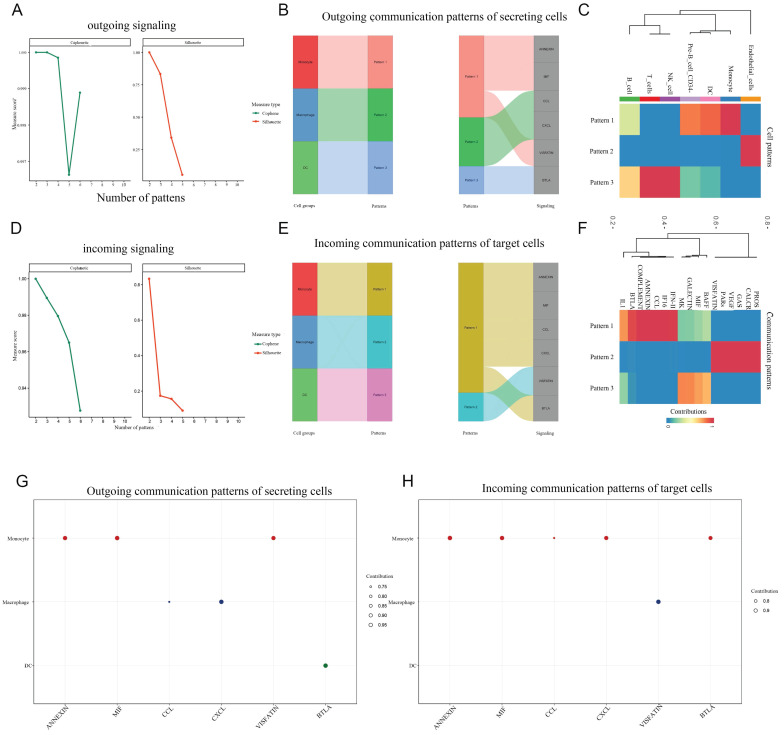
** Global communication patterns in cell subpopulations.** A,D: The communication mode. B,E: The proportion of signal pathways under different modes in the composite Sangi diagram. C,F: Cluster heatmap of global communication patterns in cell subpopulations. G,H: The signaling effects strength of different signaling pathways, outgoing and incoming.

We separately the extracted data from monocytes and DCs for cell subpopulation analysis, and then conducted cell communication analysis after dimensionality reduction clustering, providing a more refined cell communication pattern. Monocytes, as the precursors of macrophages and DCs, differentiate based on signals from their environments. It can be concluded that monocytes communicate strongly reciprocally. From the heatmap, it can be concluded that monocytes can recognize a large number of different income signals, which may be related to the state in which monocytes are striving to recognize signals from the surrounding environment. Interestingly, DC hardly recognizes incoming signals and only sends outgoing signals. We present the ligand-receptor ligation involved in monocyte-macrophage communication using a string diagram. It is worth noting that ANXA1-FPR1 is a key signal for self-communication in monocytes (Figure 7-8).

### Overexpression of ALDH2 can reduce the proliferation and migration of HCC cells

**Figure 9:**
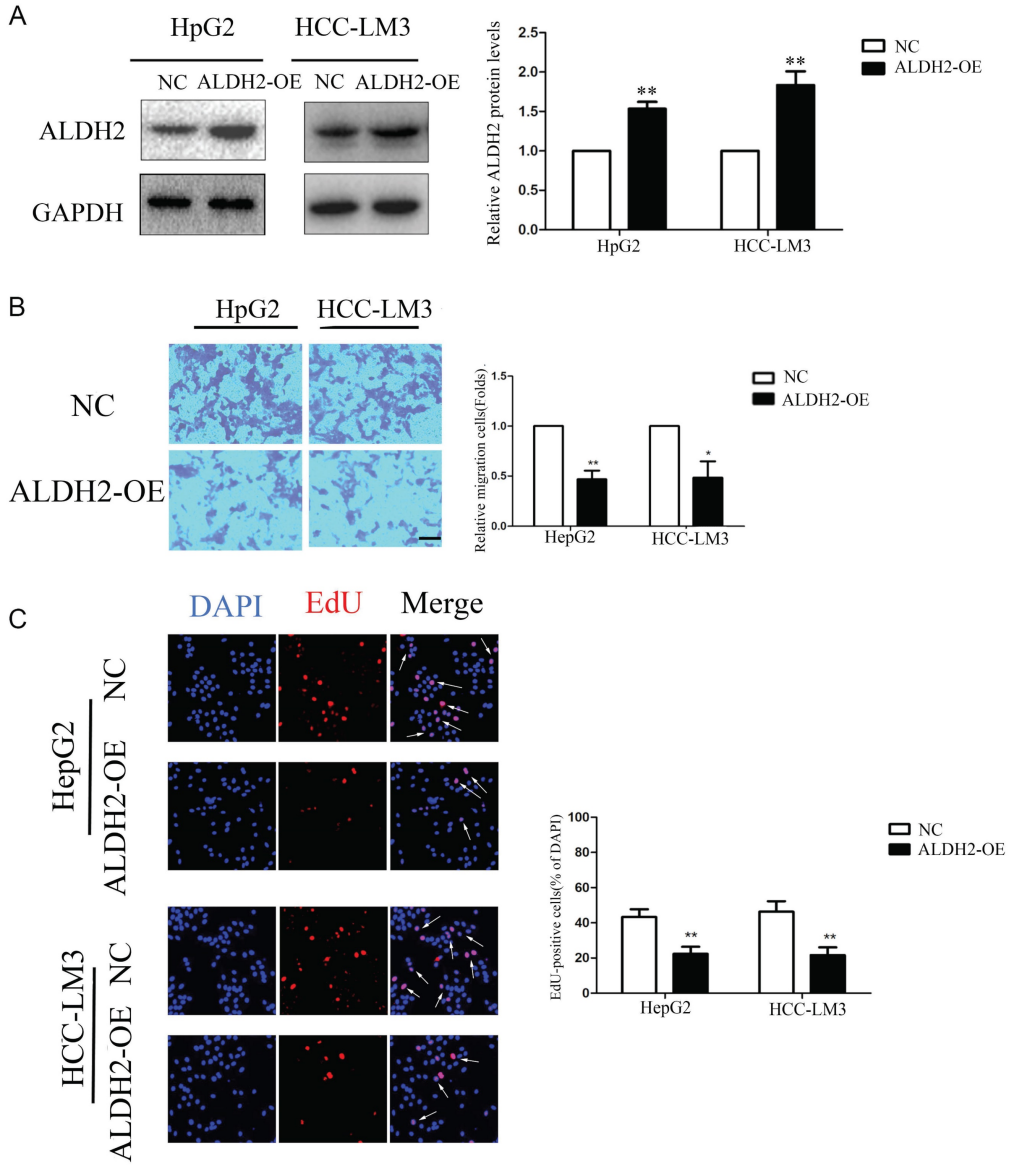
** Overexpression of ALDH2 inhibited HCC cells proliferation and migration.** A: Western blotting for the expressions of ALDH2 protein with overexpression of ALDH2. B: Transwell assay of HCC cells with overexpression of ALDH2. C: EdU immunofluorescence test of HCC cells with overexpression of ALDH2. Arrowheads indicated proliferative cells. ^*^P<0.05,^ **^P<0.01.

ALDH2 was overexpressed in HCC cells using lentivirus infection. The results of immunoblotting analysis showed that the expression levels of ALDH2 protein were significantly increased in HCC cells transfected with ALDH2 (Figure 9A). The transwell migration experiment results showed that the ALDH2 overexpression inhibited cell migration compared to control group (Figure 9B). The EdU immunofluorescence assay results showed that the positive rate of HCC cell proliferation, in the ALDH2 overexpression group, decreased compared to control group (Figure 9C).

## Discussion

HCC is a top ranked cancer and a significant cause of cancer death worldwide. In economically developed countries, the incidence rate of HCC has been increasing, making HCC a global threat to human life 2. Its prognosis is poor, and the survival rate after surgical resection is still unsatisfactory in clinical practice. Exploring biomarkers with prognostic or therapeutic value is of great significance 18-19.

The immunosuppressive tumor microenvironment caused by tumor metabolism plays an important role in the formation and development of tumors, characterized by hypoxia and acidity. Trp, as an essential amino acid has various physiological functions, such as affecting metabolism, protein synthesis, and producing various bioactive substances 20. It plays an important role in the metabolic process of tumors. Trp participates in regulating inflammatory reactions, oxidative stress, and immune activation reactions, playing a very important role in the tumor microenvironment and tumor metabolism. The formation and development of tumors often undergo changes in Trp metabolism, accompanied by abnormal expression of Trp related enzyme genes. Trp is mainly metabolized through KP, which not only promotes the inherent malignant characteristics of tumor cells but also limits tumor immunity. Therefore, it is an important drug development target for cancer immunotherapy 21.

ALDH2 is expressed in various tissues, but it is most expressed in the liver which mainly plays a role in metabolizing alcohol in the liver. As is well known, the occurrence of many tumors is closely related to alcohol, such as HCC, gastric cancer, and so on. The researches have shown that ALDH2 gene polymorphism is closely related to the occurrence of gastric cancer, esophageal cancer, and other cancers. In renal clear cell carcinoma tissue, the expression of ALDH2 is significantly lower than that in adjacent tissues, which is also reported in HCC tissue 22,23. It was shown that overexpression of ALDH2 can inhibit the invasion and migration of lung adenocarcinoma cells, and the expression of ALDH2 is related to the overall survival rate of lung cancer and HCC patients: low expression of ALDH2 indicates a low 5-year survival rate for patients 24. ALDH2 may be a potential tumor suppressor gene.

The study demonstrates that Trp metabolism-related DECs for HCC play significant roles similar to the previous reports 25-28. Other researches were just at the level of NGS, while our study uniquely analysed the single-cell sequencing data. ALDH2 expression was found to be reduced in tumor tissues, indicating its potential as a protective gene in HCC. This aligned with the previous researches suggesting the involvement of ALDH2 in various cancers and its role in metabolizing substances, including alcohol, which can influence cancers development.

We reveal a specific increase in ALDH2 expression in monocytes/macrophages. This finding is crucial as monocytes and macrophages are key players in the tumor microenvironment, contributing to tumor growth and metastasis 29. However, the specific function of ALDH2 overexpressing in these immune cells still needs further investigation. The study's findings highlight the potential of targeting ALDH2 and Trp metabolism pathways as therapeutic strategies in HCC. The therapies targeting modulation of ALDH2 activity or Trp metabolism could impact the tumor microenvironment and improve patient outcomes.

## Conclusion

In summary, our study demonstrated that ALDH2 is closely related to Trp metabolism in HCC and may serve as a potentially protective factor with an independent prognostic significance.

## Figures and Tables

**Table 1:** COX regression of Univariate analysis and Multivariate analysis

Characteristics	Total(N)	Univariate analysis		Multivariate analysis	
		Hazard ratio (95% CI)	P value	Hazard ratio (95% CI)	P value
MAOB	373	0.909 (0.774-1.068)	0.247		
CYP1A2	373	0.968 (0.909-1.032)	0.318		
KYNU	373	1.176 (1.001-1.382)	**0.049**	1.157 (0.977-1.370)	0.090
CYP2E1	373	0.961 (0.919-1.006)	0.086	0.993 (0.938-1.051)	0.814
ALDH2	373	0.715 (0.603-0.849)	**<0.001**	0.723 (0.571-0.917)	**0.007**
CYP2C18	373	1.023 (0.931-1.125)	0.632		
TDO2	373	0.998 (0.929-1.072)	0.960		
AOX1	373	0.927 (0.862-0.997)	**0.040**	0.978 (0.877-1.091)	0.692
CYP3A4	373	0.944 (0.901-0.990)	**0.018**	1.019 (0.955-1.087)	0.571
INMT	373	0.879 (0.772-1.001)	0.051	0.882 (0.775-1.005)	0.059
